# A Penalized Matrix Normal Mixture Model for Clustering Matrix Data

**DOI:** 10.3390/e23101249

**Published:** 2021-09-26

**Authors:** Jinwon Heo, Jangsun Baek

**Affiliations:** Department of Mathematics and Statistics, Chonnam National University, 77 Yongbong-ro, Buk-gu, Gwangju 61186, Korea; jwwww0124@naver.com

**Keywords:** clustering, image analysis, matrix normal distribution, expectation maximization algorithm, penalized likelihood

## Abstract

Along with advances in technology, matrix data, such as medical/industrial images, have emerged in many practical fields. These data usually have high dimensions and are not easy to cluster due to their intrinsic correlated structure among rows and columns. Most approaches convert matrix data to multi dimensional vectors and apply conventional clustering methods to them, and thus, suffer from an extreme high-dimensionality problem as well as a lack of interpretability of the correlated structure among row/column variables. Recently, a regularized model was proposed for clustering matrix-valued data by imposing a sparsity structure for the mean signal of each cluster. We extend their approach by regularizing further on the covariance to cope better with the curse of dimensionality for large size images. A penalized matrix normal mixture model with lasso-type penalty terms in both mean and covariance matrices is proposed, and then an expectation maximization algorithm is developed to estimate the parameters. The proposed method has the competence of both parsimonious modeling and reflecting the proper conditional correlation structure. The estimators are consistent, and their limiting distributions are derived. We applied the proposed method to simulated data as well as real datasets and measured its clustering performance with the clustering accuracy (ACC) and the adjusted rand index (ARI). The experiment results show that the proposed method performed better with higher ACC and ARI than those of conventional methods.

## 1. Introduction

Among the clustering methods, the Gaussian mixture model, which is model-based clustering, is widely used thanks to its easy interpretability. Since most clustering approaches suffer from the curse of dimensionality in “high dimension/low sample size data”, so does model-based clustering. The problem is further exacerbated in a Gaussian mixture model that has to estimate multiple covariance matrices. For example, given a *p*-dimensional random sample $\left\{X_{i}={\left(X_{i1},\ldots ,X_{ip}\right)}^{T},i=1,\ldots ,n\right\}$ for a multivariate Gaussian mixture model with G components, we need to estimate a total of Gp(p+1)/2+Gp+G−1 parameters. So, the number of parameters to be estimated grows quickly with dimension *p*. To address this problem, a penalized Gaussian mixture clustering model has been proposed that imposes constraints on the mean or precision matrix. In particular, Ref. [1] suggests a penalized multivariate normal mixture clustering model for variable selection by regularizing the mean structure. In each cluster, there may be some variables that are not relevant to distinguish it from other clusters. So, finding the non-informative (noise) variables and removing them may largely improve interpretability. Additionally, Ref. [2] suggests a penalized multivariate normal mixture clustering model for achieving better clustering performance by regularizing the precision matrices. In another way, Ref. [3] adopts the idea of a penalized covariance estimation from [4] and applies a lasso-type penalty to the off-diagonal component of the precision matrices.

However, as the dimension *p* increases, the computational speed extremely slows down, making it difficult to estimate the parameters practically for these methods. In recent years, along with advances in technology, matrix data have emerged in many practical fields. The conventional Gaussian mixture method converts these matrix data into vector data. So, the dimension of the data can be extremely large, i.e., a p×q matrix will be converted to a vector of pq dimension. In addition, clustering a matrix dataset is difficult because correlations between variables or occasions can change across occasions. To solve the aforementioned issues, Ref. [5] suggests finite mixtures of matrix normal distributions for matrix data. Direct analysis of three-way data structures by using a matrix normal distribution allows considering and estimating correlations between variables and occasions. Recently, the matrix mixture model was extended for various non-normal matrix data: Refs. [6,7] proposed finite mixture of matrix skewed distributions, and [8] introduced two matrix-variate distributions—both elliptical heavy-tailed generalizations of the matrix-variate normal distribution that are used in a finite mixture model. Ref. [9] suggested a penalized matrix normal mixture model by imposing a penalty for the mean matrix. This method can capture the row-wise and column-wise correlation simultaneously and has the ability to find the sparsity nature that is inherent in the signals and images. However, the approach of [9] shows weakness for high dimension/low sample size data because it cannot reduce the number of precision parameters to estimate.

In a multivariate normal distribution, the conditional correlation between two variables, given the other variables, is explicitly determined by the precision matrix. That is, if the (i,j)th element of the precision matrix is zero, then the conditional correlation between the *i*th and the *j*th variables given the other variables is zero, and they are conditionally independent. Ref. [10] introduced the idea of covariance selection, which consists of inferring a sparse estimation of the precision matrix and interpreting its sparsity structure as a conditional dependency between variables. A Gaussian graphical model is a network whose values on the vertices follow a centered multivariate normal distribution, and has been applied to analyze high-dimensional data with small sample size through the regularized estimation of the precision matrix [11]. A notable approach for the precision matrix estimation is the graphical lasso algorithm [12].

In this paper, the work of [9] is extended by further regularizing the precision matrix as in the Gaussian graphical model, to cope better with the curse of dimensionality for large size images. Specifically, we propose a penalized matrix normal mixture model with lasso-type penalty terms in both mean and covariance matrices, and then an expectation maximization algorithm is developed to estimate the parameters. The estimators are consistent and their limiting distributions are derived. The experiment results show that the proposed approach attains much improved clustering performance in comparison with conventional methods.

The capability of the proposed model is compared with two popular distance-based methods and two conventional model-based methods in Appendix A Table A1. The major contributions of this research to overcome the limitations of the conventional methods are as follows:

(1) Since the data type for the Gaussian mixture model is a vector and K-means and spectral clustering methods use dissimilarity and similarity between the observations as input, these methods do not have the modeling ability for the row-wise/column-wise covariance structure of matrix data (images). On the other hand, both [9] and the proposed model can capture the row-wise and column-wise correlation of image pixels simultaneously by estimating the row covariance *U* and column covariance *V*, where the covariance matrix Σ is the Kronecker product of *U* and *V*. This capability of modeling separable row-wise/column-wise covariance structure helps for better clustering of image data.
(2) Because the proposed model is a model-based clustering method, it has the sound mathematical basis and the interpretability of results with the estimated parameters, whereas centroid-based and similarity-based methods do not.
(3) If variables are measured on different scales and one variable has much larger values than the others, then the first variable will dominate the clustering. Therefore, it is preferred to use a clustering method that is invariant against affine transformations. K-means and similarity-based methods using the Euclidean distance are not invariant against affine transformations, whereas Gaussian mixture models are.
(4) Model-based clustering methods have free parameters to estimate and often have difficulty in the estimation of parameters when the data are of a high dimension but the sample is small. Given p×q, random matrix data *X*, and the number of clusters *G*, the conventional Gaussian mixture model needs to estimate a total of Gpq(pq+1)/2+Gpq+G−1 parameters because it converts the data into a vector of pq dimension. The method [9] is regularized on the mean matrix, and thus it needs to estimate Gp(p+1)/2+Gq(q+1)/2+Gpq+G−1−r parameters, where *r* is the number of the mean parameters values that are shrunken to zero. Since the proposed method is regularized further on the precision matrix, that is, on the row and column covariances, *U* and *V*, with shrunken elements, it needs to estimate Gp(p+1)/2+Gq(q+1)/2+Gpq+G−1−r* parameters, where r* is the number of the mean and precision parameters values that are shrunken to zero. The range of *r* and $r^{*}$ is 0≤r≤Gpq and r≤r*≤Gp(p+1)/2+Gq(q+1)/2+Gpq, respectively. Therefore, the proposed approach is a more parsimonious model that has the fewest parameters and is able to cope better with the curse of dimensionality for large size images.
(5) There often exist correlations between the rows and the columns of matrix data. A separable covariance structure of the proposed model can effectively reduce parameters, speed up calculations, and provide practical interpretability. Therefore, the proposed method has the competence of both parsimonious modeling to overcome the curse of dimensionality and reflecting the proper conditional correlation structure for high dimension/low sample size data in comparison with the conventional methods.

The paper is organized as follows. In Section 2, the matrix normal mixture model is explained and then the penalized matrix normal mixture model with the mean and precision matrices is introduced. Next, a new EM algorithm for the precision matrices of the proposed model, a model selection method, k-fold cross-validation, and the asymptotic theory are developed. In Section 3, the proposed method is applied to both simulation data and real data. In the simulation data experiment, the clustering performance is compared with that of the K-means clustering method [13], the spectral clustering method [14], and [9]’s method. The consistency of the estimates for the proposed model is shown, using the averaged spectral norm of the difference (SL) and the averaged Frobenius norm of the difference (FL) [3]. In the real data experiment, the clustering performance is also compared with that of the K-means clustering method, the spectral clustering method and the method of [9]. In Section 4, we summarize the results and discuss future work. The Appendix A, Appendix B and Appendix C provides a comparative overview table and the proofs of the penalized maximum likelihood estimator of the mean matrix for the proposed model and the asymptotic theory.

## 2. Method

A mixture model using matrix normal distribution was previously introduced by [5]. The matrix normal mixture model [15,16,17] is reviewed is shown in Section 2.1. Then, our penalized matrix normal mixture model, estimation, asymptotic theory, and model selection are presented in Section 2.2, Section 2.3 and Section 2.4.

### 2.1. Matrix Normal Mixture Model (MNMM)

#### 2.1.1. Definition of MNMM

The p×q random matrix *X* has a matrix normal distribution, with mean matrix *M* and row covariance *U* and column covariance *V*, denoted by $X∼N_{p,q}\left(M,U,V\right)$, if the p.d.f. of *X* is given by the following:

(1) $$f\left(X|M,U,V\right)={\left(2\pi \right)}^{-\frac{pq}{2}}{\left|V\right|}^{-\frac{p}{2}}{\left|U\right|}^{-\frac{q}{2}}exp\left\{-\frac{1}{2}tr\left(V^{-1}{\left(X-M\right)}^{T}U^{-1}\left(X-M\right)\right)\right\},$$

where $M\in R^{p\times q},U\in R^{p\times p},V\in R^{q\times q}$ and |A|,tr(A) are the determinant value and the trace of a matrix *A*, respectively. The p.d.f. (1) can be converted to the p.d.f. of the vectorization of *X* as follows:

$$vec\left(X\right)∼N_{pq}\left(vec\left(M\right),V⊗U\right),$$

where vec is the vectorization operator and ⊗ is the Kronecker product. The matrix normal distribution is a generalization of the matrix variate, i.e., it can effectively handle three-way data. In particular, the separable covariance structure has an important advantage, which is reducing the number of parameters. For example, given the p×q random matrix *X*, the dimension of inseparable covariance matrix Σ is pq×pq. However, the dimension of separable covariance matrix Σ=V⊗U is p×p+q×q.

#### 2.1.2. Maximum Likelihood Estimation

The log-likelihood function for given i.i.d observations $X_{1},X_{2},\ldots ,X_{n}$ of $N_{p,q}\left(M,U,V\right)$ is as follows:

$$\begin{array}{cc} L(M,U,V;X)= & -\frac{npq}{2}log2\pi -\frac{np}{2}log|V|-\frac{nq}{2}log\left|U\right|\\ \; & -\frac{1}{2}\sum_{i=1}^{n}tr\left\{(V^{-1}{\left(X_{i}-M\right)}^{T}U^{-1}\left(X_{i}-M\right)\right\}. \end{array}$$

The maximum likelihood estimators (m.l.e.) for *M*, *U*, *V* are as follows:

(2) $$\begin{array}{cc} \; & \hat{M}=\bar{X},\\ \; & {\hat{U}}^{\left(t+1\right)}=\frac{1}{nq}\sum_{i=1}^{n}\left(X_{i}-\bar{X}\right){\left({\hat{V}}^{\left(t\right)}\right)}^{-1}{\left(X_{i}-\bar{X}\right)}^{T},\\ \; & {\hat{V}}^{\left(t+1\right)}=\frac{1}{np}\sum_{i=1}^{n}{\left(X_{i}-\bar{X}\right)}^{T}{\left({\hat{U}}^{\left(t+1\right)}\right)}^{-1}\left(X_{i}-\bar{X}\right). \end{array}$$

Note that m.l.e., $\hat{U}$ and $\hat{V}$ are of the no closed-form solution, and $a\hat{V}$ and $b\hat{U}$, which is a constant multiple of $\hat{U}$ and $\hat{V}$, are also solutions. However, $a\hat{V}⊗b\hat{U}$ must be the same as $\hat{V}⊗\hat{U}$ [15]. $\hat{U}$ and $\hat{V}$ can be obtained by the iterative algorithm of (2) until the convergence criterion is satisfied. The criterion is set as $∥{\hat{U}}^{\left(t\right)}-{\hat{U}}^{\left(t+1\right)}{∥}_{F}/{∥{\hat{U}}^{\left(t\right)}∥}_{F}<0.01$ and $∥{\hat{V}}^{\left(t\right)}-{\hat{V}}^{\left(t+1\right)}{∥}_{F}/{∥{\hat{V}}^{\left(t\right)}∥}_{F}<0.01$.

#### 2.1.3. EM Algorithm of MNMM

Given i.i.d p×q observations $X_{1},X_{2},\ldots ,X_{n}$ from the mixture of matrix normal distributions with *G* groups, the p.d.f. of the matrix normal mixture is as follows:

$$g\left(X\right)=\sum_{j=1}^{G}\pi_{j}f\left(X|\Theta_{j}\right),$$

where $\Theta_{j}$ is the set of parameters corresponding to the *j*th group, i.e., $\Theta_{j}=\left(M_{j},U_{j},V_{j}\right)$, $\pi_{1},\ldots ,\pi_{G}$ are the weights of belonging to the group *g*, and *f* is the matrix normal distribution with mean $M_{j}$, row covariance $U_{j}$, column covariance $V_{j}$. Then, the log-likelihood function of MNMM is as follows:

(3) $$L\left(\Theta ;X\right)=\sum_{i=1}^{n}log\left\{\sum_{j=1}^{G}\pi_{j}f\left(X_{i}|\Theta_{j}\right)\right\}.$$

It is difficult to obtain the maximum log-likelihood value by solving the first derivative Equation (3) because the p.d.f is defined as the sum in logarithm. So, the EM algorithm is widely used to estimate parameters by maximizing the conditional expectation for the latent variable z, given the data instead of the log-likelihood function [18].

In the E-step, the posterior probability of the latent variables $z_{ij}$ is defined by Bayes’ theorem as follows:

(4) $${\hat{\tau }}_{ij}^{\left(t\right)}=\frac{{\hat{\pi }}_{j}^{\left(t\right)}f\left(X_{i}|{\hat{\Theta }}_{j}^{\left(t\right)}\right)}{\sum_{l=1}^{G}{\hat{\pi }}_{l}^{\left(t\right)}f\left(X_{i}|{\hat{\Theta }}_{l}^{\left(t\right)}\right)}.$$

In the M-step, the parameters, ${\hat{\Theta }}^{\left(t+1\right)}$, that maximize the expected conditional log-likelihood given data are found as follows:

$${\hat{\Theta }}^{\left(t+1\right)}=\underset{{\hat{\Theta }}_{j}}{argmax}\sum_{i=1}^{n}\sum_{j=1}^{G}{\hat{\tau }}_{ij}^{\left(t\right)}log\left\{\pi_{j}f\left(X_{i}|\Theta_{j}\right)\right\}.$$

We obtain the following estimates by using the partial derivative and some algebra.

$$\begin{array}{cc} \; & {\hat{\pi }}_{j}^{\left(t+1\right)}=\frac{\sum_{i=1}^{n}{\hat{\tau }}_{ij}^{\left(t\right)}}{n},\;{\hat{M}}_{j}^{\left(t+1\right)}=\frac{\sum_{i=1}^{n}{\hat{\tau }}_{ij}^{\left(t\right)}X_{i}}{\sum_{i=1}^{n}{\hat{\tau }}_{ij}^{\left(t\right)}},\\ \; & {\hat{U}}_{j}^{\left(t+1\right)}=\frac{\sum_{i=1}^{n}{\hat{\tau }}_{ij}^{\left(t\right)}\left(X_{i}-{\hat{M}}_{j}^{\left(t+1\right)}\right){\left({\hat{V}}_{j}^{\left(t\right)}\right)}^{-1}{\left(X_{i}-{\hat{M}}_{j}^{\left(t+1\right)}\right)}^{T}}{q\sum_{i=1}^{n}{\hat{\tau }}_{ij}},\\ \; & {\hat{V}}_{j}^{\left(t+1\right)}=\frac{\sum_{i=1}^{n}{\hat{\tau }}_{ij}^{\left(t\right)}{\left(X_{i}-{\hat{M}}_{j}^{\left(t+1\right)}\right)}^{T}{\left({\hat{U}}_{j}^{\left(t+1\right)}\right)}^{-1}\left(X_{i}-{\hat{M}}_{j}^{\left(t+1\right)}\right)}{r\sum_{i=1}^{n}{\hat{\tau }}_{ij}}. \end{array}$$

Similar to the m.l.e. of the matrix normal distribution, ${\hat{U}}_{j}$ and ${\hat{V}}_{j}$ are obtained by updating the previously estimated parameters in the EM algorithm until a convergence criterion is satisfied.

### 2.2. Penalized Matrix Normal Mixture Model (PMNMM)

Let $M_{g(k,l)}$ be the (k,l)th element of the mean matrix of group *g*, $M_{g}$. If $M_{g(k,l)}$ is the same for all groups, i.e., $M_{1(k,l)}=\ldots \;=M_{G(k,l)}$, then the (k,l)th element of the mean matrix cannot differentiate the clusters. In this case, when the data are standardized, the (k,l)th element of the standardized mean matrix of each cluster will be 0, and is known to be the noise variable (non-informative) for clustering [2]. Therefore, penalizing on the elements of mean matrix of the matrix normal mixture was proposed by [9] for identifying the underlying spatial structure and improving the signal-to-noise ratio (SNR) of the local field potentials (LFP) signals. However, in order to improve the clustering performance in high-dimension/low sample size data, the regularization of the precision matrix is inevitable because the number of parameters required to estimate the multiple covariance matrices becomes very large. In this paper, the method of [9] is extended by further regularizing the precision matrix to cope better with the curse of dimensionality and reflect the proper conditional correlation structure for matrix data.

#### 2.2.1. Penalized Log-Likelihood Function of PMNMM

The penalized log-likelihood function is considered as follows:

$$L_{p}\left(\Theta ;\Lambda ,X\right)=\sum_{i=1}^{n}log\left[\sum_{j=1}^{G}\pi_{j}f\left(X_{i}|\Theta_{j}\right)\right]-\lambda_{1}\sum_{j=1}^{G}|M_{j}{|}_{1}-\lambda_{2}\sum_{j=1}^{G}|U_{j}^{-1}{|}_{1}-\lambda_{3}\sum_{j=1}^{G}{\left|V_{j}^{-1}\right|}_{1},$$

where $\lambda_{1},\lambda_{2},\lambda_{3}>0$ are the tuning parameters, and ${\left|A\right|}_{1}$ is the L1 norm, which is the sum of all absolute values of elements of A. Setting $\lambda_{2},\lambda_{3}=0$ is equivalent to the penalized matrix normal mixture model with lasso regularization of the mean matrix proposed in [9]. Additionally, setting $\lambda_{1},\lambda_{2},\lambda_{3}=0$ is equivalent to the non-regularized matrix mixture model proposed in [5].

#### 2.2.2. EM Algorithm of PMNMM

The EM algorithm is employed as in Section 2.1.3. The E-step is the same as Equation (4). In the M-step, the parameter estimates can be obtained by solving the constraint optimization problem as follows:

$${\tilde{\Theta }}^{\left(t+1\right)}=\underset{M_{j},U_{j}\&V_{j}>0}{argmax}\left\{L\left(\Theta ;X\right)-\lambda_{1}\sum_{j=1}^{G}|M_{j}{|}_{1}-\lambda_{2}\sum_{j=1}^{G}|U_{j}^{-1}{|}_{1}-\lambda_{3}\sum_{j=1}^{G}{\left|V_{j}^{-1}\right|}_{1}\right\},$$

where $U_{j}\&V_{j}>0$ means that $U_{j}$ and $V_{j}$ are symmetric and positive definite matrices. The estimate of *M* can be obtained by the subgradient approach as shown in Appendix B as follows:

(5) $${\left({\tilde{M}}_{j}^{\left(t+1\right)}\right)}_{\left(k,l\right)}=sign{\left({\hat{M}}_{j}^{\left(t\right)}\right)}_{\left(k,l\right)}{\left[abs\left({\hat{M_{j}}}^{\left(t\right)}\right)-\frac{\lambda_{1}}{\sum_{i=1}^{n}\tau_{ij}}{\hat{U_{j}}}^{\left(t\right)}1_{p\times q}{\hat{V_{j}}}^{\left(t\right)}\right]}_{(k,l)+},\;j=1,\ldots ,G,$$

where ${\hat{M_{j}}}^{\left(t\right)}=\frac{\sum_{i=1}^{n}{\hat{\tau }}_{ij}X_{i}}{\sum_{i=1}^{n}{\hat{\tau }}_{ij}}$ is the m.l.e. of MNMM, ${\left[A\right]}_{(k,l)+}=max\left(A_{\left(k,l\right)},0\right)$, $1_{p\times q}$ is p×q the matrix with all components of 1, and $sign{\left(A\right)}_{\left(k,l\right)}$ is the function that extracts the sign of a real number, and abs(A) is the absolute value of *A*. Then, we focus on the constraint optimization problem with respect to each *U* and *V*. To maximize the log-likelihood with the constraint, the problem is redefined as the constraint optimization with respect to $U_{j}^{-1}$ and $V_{j}^{-1}$ as follows:

(6) $$\underset{U_{j}>0}{argmax}\sum_{j=1}^{G}\left\{\frac{q\sum_{i=1}^{n}{\hat{\tau }}_{ij}^{\left(t\right)}}{2}log|U_{j}^{-1}|-\frac{q\sum_{i=1}^{n}{\hat{\tau }}_{ij}^{\left(t\right)}}{2}tr\left({\tilde{S}}_{j}^{\left(U\right)}U_{j}^{-1}\right)-\lambda_{2}\sum_{j}{\left|U_{j}^{-1}\right|}_{1}\right\}$$

with

(7) $${\tilde{S}}_{j}^{\left(U\right)}=\frac{\sum_{i=1}^{n}{\hat{\tau }}_{ij}^{\left(t\right)}\left(X_{i}-{\tilde{M}}_{j}^{\left(t\right)}\right){\left({\hat{V}}_{j}^{-1}\right)}^{\left(t\right)}{\left(X_{i}-{\tilde{M}}_{j}^{\left(t\right)}\right)}^{T}}{q\sum_{i=1}^{n}{\hat{\tau }}_{ij}^{\left(t\right)}}$$

and

(8) $$\underset{V_{j}>0}{argmax}\sum_{j=1}^{G}\left\{\frac{p\sum_{i=1}^{n}{\hat{\tau }}_{ij}^{\left(t\right)}}{2}log|V_{j}^{-1}|-\frac{p\sum_{i=1}^{n}{\hat{\tau }}_{ij}^{\left(t\right)}}{2}tr\left({\tilde{S}}_{j}^{\left(V\right)}V_{j}^{-1}\right)-\lambda_{3}\sum_{j}{\left|V_{j}^{-1}\right|}_{1}\right\}$$

with

(9) $${\tilde{S}}_{j}^{\left(V\right)}=\frac{\sum_{i=1}^{n}{\hat{\tau }}_{ij}^{\left(t\right)}{\left(X_{i}-{\tilde{M}}_{j}^{\left(t\right)}\right)}^{T}{\left({\hat{U}}_{j}^{-1}\right)}^{\left(t+1\right)}\left(X_{i}-{\tilde{M}}_{j}^{\left(t\right)}\right)}{p\sum_{i=1}^{n}{\hat{\tau }}_{ij}^{\left(t\right)}}.$$

However, for Equations (6)–(9), the subgradient approach is not possible because the solution of the constraint optimization problem must satisfy the conditions that $U_{j}$ and $V_{j}$ are symmetric and positive definite matrices. So, the graphical lasso algorithm [12] is applied to maximize the following constraint optimization function with respect to positive definite matrix Σ.

$$log|\Sigma^{-1}|-tr\left(S\Sigma^{-1}\right)-\lambda {\left|\Sigma^{-1}\right|}_{1},$$

where *S* is the sample covariance matrix. Therefore, we can apply the algorithm by substituting $S={\tilde{S}}_{j}^{\left(U\right)}$, Σ=U, $\lambda =2\lambda_{2}/q\sum_{i=1}^{n}{\hat{\tau }}_{ij}^{\left(t\right)}$ and $S={\tilde{S}}_{j}^{\left(V\right)}$, Σ=V, $\lambda =2\lambda_{3}/p\sum_{i=1}^{n}{\hat{\tau }}_{ij}^{\left(t\right)}$ to estimate $U_{j}^{-1}$ and $V_{j}^{-1}$, respectively. The graphical lasso algorithm is implemented with R package glasso [12]. Then the m.l.e. of $U^{-1}$ and $V^{-1}$ are obtained by iterating E-step and M-step until the penalized log-likelihood converges. To prevent the convergence of the parameter estimation from prematurely stopping, Aitken’s acceleration [19] is used. Aitken’s acceleration computes the acceleration coefficient at iteration t as follows:

$$a^{\left(t\right)}=\frac{l^{\left(t\right)}-l^{\left(t-1\right)}}{l^{\left(t-1\right)}-l^{\left(t-2\right)}},$$

where $l^{\left(t\right)}$ is the penalized log-likelihood value at iteration *t*. The accelerated maximum of the penalized log-likelihood at iteration *t* is as follows:

$${\hat{l}}^{\left(t\right)}=l^{\left(t-1\right)}+\frac{l^{\left(t\right)}-l^{\left(t-1\right)}}{1-a^{\left(t\right)}}.$$

The iteration stops when $0\leq \left({\hat{l}}^{\left(t\right)}-l^{\left(t\right)}\right)/\left|l^{\left(t\right)}\right|\leq \epsilon$ for very small positive number ϵ. We set ϵ=0.001.

The iterative parameter estimation procedure needs the initial starting values. So the K-means clustering method is used to obtain the initial parameter values. For the fixed number of clusters G, the vectorized data are divided into G clusters, using K-means. Then, based on a group label that is predicted, the m.l.e. are obtained for each cluster; we use those values as the initial value set for the EM algorithm. Additionally, in the process of estimating the parameters that maximize the objective function, the EM algorithm has to be run multiple times because local maximum values may exist.

### 2.3. Asymptotic Theory

The consistency of the penalized likelihood estimator for the mean of the mean-regularized matrix normal mixture model [9] under the following mild conditions was shown by the direct application of Theorem 1 in [20]. That is, the parameter space $\Psi^{d_{1},d_{2}}$ must be defined as follows:

(10) $$\begin{array}{cc} \Psi^{d_{1},d_{2}} & =\{\left(\pi_{1},\ldots ,\pi_{G},M_{1},\ldots ,M_{G},V_{1}⊗U_{1},\ldots ,V_{G}⊗U_{G}\right)\in \Psi^{d_{1},d_{2}}:\frac{\sigma_{i}\left(U_{h}\right)}{\sigma_{i}\left(V_{h}\right)}=c_{h}\\ \; & for\;i=1,\ldots ,min\left\{p,q\right\}\;and\;h=1,\ldots ,G\} \end{array}$$

to guarantee the identifiability of the matrix normal mixture model.

Since Theorem 1 in [20] is applicable to all lasso type penalized likelihood estimators, the penalized likelihood estimators of the proposed model are also consistent under the condition (10). We further establish the asymptotic distributions of the estimators, and prove their consistency in another way. We derive asymptotic distributions of the proposed estimators, using the techniques in [4]. The proof is given in Appendix C.

**Theorem** **1.** Let $X_{1},\ldots ,X_{n}$ be a random sample from a mixture matrix normal distribution. Let ${\tilde{M}}_{j}$, ${\tilde{U}}_{j}^{*}$, and ${\tilde{V}}_{j}^{*}$ be the penalized maximum likelihood estimator of $M_{j}$, $U_{j}^{-1}$, and $V_{j}^{-1}$, respectively, j=1,…,G. Assume that the true parameter value $M_{j},U_{j},V_{j}\in {\bar{\Psi }}^{d_{1},d_{2}}$, and $\sqrt{n}\lambda_{i}\to \lambda_{i0}\geq 0$ as n→∞, i=1,2,3; then,

*(1) $\sqrt{n}\left({\tilde{M}}_{j}-M_{j}\right)\overset{d}{\to }\underset{H}{argmin}\left(V_{1}\right),$*

*where*

$$\begin{array}{cc} V_{1}\left(H\right)= & -tr\left(U_{j}^{-1}HV_{j}^{-1}W_{1}^{T}\right)-tr\left(V_{j}^{-1}H^{T}U_{j}^{-1}W_{1}\right)+tr\left(V_{j}^{-1}H^{T}U_{j}^{-1}H\right)\\ \; & +\lambda_{10}\sum_{l}\sum_{m}\left\{H\left(l,m\right)sign\left({\tilde{M}}_{j}\left(l,m\right)\right)I\left({\tilde{M}}_{j}\left(l,m\right)\neq 0\right)+\left|H\left(l,m\right)\right|I\left({\tilde{M}}_{j}\left(l,m\right)=0\right)\right\}, \end{array}$$

*in which H is a p×q matrix, H(l,m) is the (l,m)th element of H, $W_{1}$ is a random p×q matrix such that $vec\left(W_{1}\right)∼N\left(0,\Lambda_{1}\right)$, and $\Lambda_{1}$ is such that the following holds:*

$$cov\left(W_{1}\left(i,j\right),W_{1}\left(i^{\prime },j^{\prime }\right)\right)=cov\left(X\left(i,j\right)-M_{j}\left(i,j\right),X\left(i^{\prime },j^{\prime }\right)-M_{j}\left(i^{\prime },j^{\prime }\right)\right),$$

(2) $\sqrt{n}\left({\tilde{U}}_{j}^{*}-U_{j}^{-1}\right)\overset{d}{\to }\underset{L=L^{\prime }}{argmin}\left(V_{2}\right)$,

*where*

$$\begin{array}{cc} V_{2}\left(L\right)= & tr\left(LU_{j}LU_{j}\right)+tr\left(LW_{2}\right)\\ \; & +\lambda_{20}\sum_{l\neq m}\left\{L\left(l,m\right)sign\left({\tilde{U}}_{j}^{*}\left(l,m\right)\right)I\left({\tilde{U}}_{j}^{*}\left(l,m\right)\neq 0\right)+\left|L\left(l,m\right)\right|I\left({\tilde{U}}_{j}^{*}\left(l,m\right)=0\right)\right\}, \end{array}$$

*in which L is a p×p matrix, L(l,m) is the (l,m)th element of L, $W_{2}$ is a random p×p matrix such that $vec\left(W_{2}\right)∼N\left(0,\Lambda_{2}\right)$, and $\Lambda_{2}$ is such that the following holds:*

$$cov\left(W_{2}\left(i,j\right),W_{2}\left(i^{\prime },j^{\prime }\right)\right)=cov\left\{\left(x_{i}^{*}-m_{i}^{*}\right)V_{j}^{-1}{\left(x_{j}^{*}-m_{j}^{*}\right)}^{T}/q,\left(x_{i^{\prime }}^{*}-m_{i^{\prime }}^{*}\right)V_{j}^{-1}{\left(x_{j^{\prime }}^{*}-m_{j^{\prime }}^{*}\right)}^{T}/q\right\},$$

where $x_{i}^{*}$ and $m_{i}^{*}$ is the ith row of the matrix X and $M_{j}$, respectively,

(3) $\sqrt{n}\left({\tilde{V}}_{j}^{*}-V_{j}^{-1}\right)\overset{d}{\to }\underset{R=R^{\prime }}{argmin}\left(V_{3}\right)$,

*where*

$$\begin{array}{cc} V_{3}\left(R\right)= & tr\left(RV_{j}RV_{j}\right)+tr\left(RW_{3}\right)\\ \; & +\lambda_{30}\sum_{l\neq m}\left\{R\left(l,m\right)sign\left({\tilde{V}}_{j}^{*}\left(l,m\right)\right)I\left({\tilde{V}}_{j}^{*}\left(l,m\right)\neq 0\right)+\left|R\left(l,m\right)\right|I\left({\tilde{V}}_{j}^{*}\left(l,m\right)=0\right)\right\}, \end{array}$$

*in which R is a q×q matrix, R(l,m) is the (l,m)th element of R, $W_{3}$ is a random q×q matrix such that $vec\left(W_{3}\right)∼N\left(0,\Lambda_{3}\right)$, and $\Lambda_{3}$ is such that the following holds:*

$$cov\left(W_{3}\left(i,j\right),W_{3}\left(i^{\prime },j^{\prime }\right)\right)=cov\left\{{\left(x_{i}-m_{i}\right)}^{T}U_{j}^{-1}\left(x_{j}-m_{j}\right)/p,{\left(x_{i^{\prime }}-m_{i^{\prime }}\right)}^{T}U_{j}^{-1}\left(x_{j^{\prime }}-m_{j^{\prime }}\right)/p\right\},$$

*where $x_{i}$ and $m_{i}$ is the ith column of the matrix X and $M_{j}$, respectively.*

**Corollary** **1.** Assume that $M_{j},U_{j},V_{j}\in {\bar{\Psi }}^{d_{1},d_{2}}$ and $\sqrt{n}\lambda_{i}\to \lambda_{i0}\geq 0$ as n→∞, i=1,2,3, then ${\tilde{M}}_{j}$, ${\tilde{U}}_{j}^{*}$, and ${\tilde{V}}_{j}^{*}$ are consistent.

**Proof.**  Since $\sqrt{n}\left({\tilde{M}}_{j}-M_{j}\right)$ converges in a distribution, $\sqrt{n}\left({\tilde{M}}_{j}-M_{j}\right)=O_{p}\left(1\right)$ and $|{\tilde{M}}_{j}-M_{j}{|}_{1}=O_{p}\left(1/\sqrt{n}\right)=o_{p}\left(1\right)$ as n→∞. Therefore, the proposed estimator ${\tilde{M}}_{j}$ is $\sqrt{n}$ consistent. The proof for ${\tilde{U}}_{j}^{*}$ and ${\tilde{V}}_{j}^{*}$ is same as for ${\tilde{M}}_{j}$.  □

### 2.4. Model Selection

**Figure 1:**
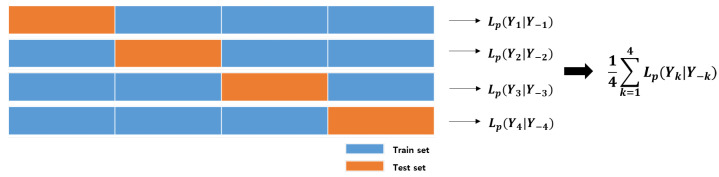
4-fold cross-validation.

In the penalized mixture model, the selection of the optimal hyperparmeters is very important. The proposed penalized matrix normal mixture model has four hyperparameters $\left(G,\lambda_{1},\lambda_{2},\lambda_{3}\right)$. A grid search method is used to select the optimal set of $\left(G,\lambda_{1},\lambda_{2},\lambda_{3}\right)$ with the largest penalized log-likelihood. However, even if the grid search method is applied, a model fitted with a fixed dataset is often not reliable due to the overfitting problem. Generally, the k-fold cross-validation (CV) method is used to solve these problems. In k-fold CV, the data $X=\left\{X_{1},X_{2},\ldots ,X_{n}\right\}$ are split into K mutually exclusive subsets denoted by $Y_{1},Y_{2},\ldots ,Y_{K}$. Then, one of the K subsets is selected as the test data, the model with the remaining data is applied, and the penalized log-likelihood value for the test data is calculated with the estimated parameters. After using each of the subsets as test data in turn, the final score is obtained by averaging the K penalized log-likelihood calculated values as follows:

$$L_{p}\left(K\right)=\frac{1}{K}\sum_{k=1}^{K}L_{p}\left(Y_{k}|Y_{-k}\right),$$

where $Y_{-k}$ is the dataset excluding $Y_{k}$. For example, in 4-fold cross-validation, the mean of the penalized log-likelihood is obtained as shown in Figure 1.

Therefore, using the k-fold cross-validation method, we can select the optimal parameter set with the largest mean of the penalized log-likelihood among different combinations of grid parameter values.

## 3. Experiments

### 3.1. Simulation Studies

**Figure 2:**
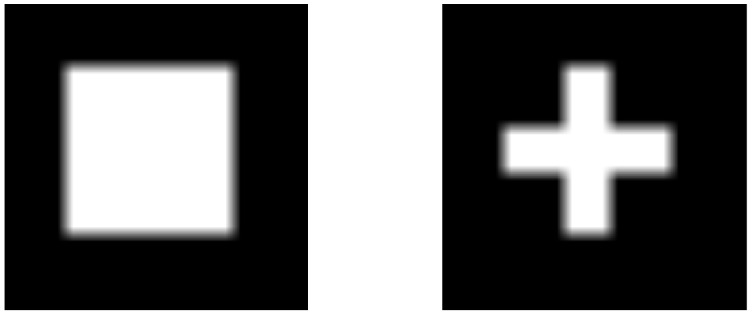
Mean structure of each group.

In Scenarios 1 and 2, we generate 20×20 data from two matrix normal distributions $N_{400}\left(vec\left(M_{1}\right),V_{1}⊗U_{1}\right),N_{400}\left(vec\left(M_{2}\right),V_{2}⊗U_{2}\right)$, each with a different type of mean structure. One has a rectangle shape and the other has a cross shape, as in Figure 2.

We fix G = 2 in the simulation study, and the ranges of the tuning parameter $\lambda_{1},\lambda_{2},\lambda_{3}$ are set to be $\lambda_{1}=20,10,5,1,0.5,0.1$, $\lambda_{2}=1,0.1,0.01,0.001$, $\lambda_{3}=1,0.1,0.01,0.001$, and the optimal $\left(\lambda_{1},\lambda_{2},\lambda_{3}\right)$ set is determined with the 4-fold CV method. The sample size *n* is set to be 100, 200, 300, and 1000. To compare the performance for different degrees of sparsity, we consider different precision structures for Scenario 1 and Scenario 2, banding and AR(1) model, respectively:

Scenario 1: Banding precision structure

$$\left(V_{1}⊗U_{1}\right)=\Sigma_{1}^{-1}=\left\{\begin{array}{cc} 1 & if\;i=j\\ 0.2 & \;if\;|i-j|=1\\ 0 & \;otherwise \end{array}\right.,\;1\leq i,j\leq 400.$$

$$\left(V_{2}⊗U_{2}\right)=\Sigma_{2}^{-1}=\left\{\begin{array}{cc} 1 & if\;i=j\\ 0.2 & \;if\;|i-j|=1\\ 0.3 & \;if\;|i-j|=2\\ 0 & \;otherwise \end{array}\right.,\;1\leq i,j\leq 400.$$

Scenario 2: AR(1) precision structure

$$\left(V_{1}⊗U_{1}\right)=\Sigma_{1}^{-1}=0.5^{\left|i-j\right|},\;1\leq i,j\leq 400.$$

$$\left(V_{2}⊗U_{2}\right)=\Sigma_{2}^{-1}=0.4^{\left|i-j\right|},\;1\leq i,j\leq 400.$$

The proposed model is compared with the conventional methods: K-means, spectral clustering, and the model of [9]. The criteria for performance are ACC and adjusted rand index (ARI) [21]. ACC shows the closeness of a predicted value to a standard or known value, and ARI is a clustering evaluation method that indicates perfect clustering if it is 0 and random clustering if it is 0. Additionally, the two norms are considered to show the consistency of the parameter estimates of the model: the averaged spectral norm and Frobenius norm of the difference between the estimated precision matrix and the truth.

$$\begin{array}{cc} SL & =\frac{1}{G}\sum_{j=1}^{G}{∥{\hat{\Sigma }}_{j}^{-1}-\Sigma_{j}^{-1}∥}_{s},\\ FL & =\frac{1}{G}\sum_{j=1}^{G}{∥{\hat{\Sigma }}_{j}^{-1}-\Sigma_{j}^{-1}∥}_{F}\\ \; & =\frac{1}{G}\sum_{j=1}^{G}\sqrt{\sum_{i}\sum_{j}{\left({\hat{\Sigma }}_{j}^{-1}\left(i,j\right)-\Sigma_{j}^{-1}\left(i,j\right)\right)}^{2}}, \end{array}$$

where ${∥A∥}_{s}$ is the largest singular value of matrix *A*, and ${∥A∥}_{F}$ is the Frobenius norm.

**Figure 3:**
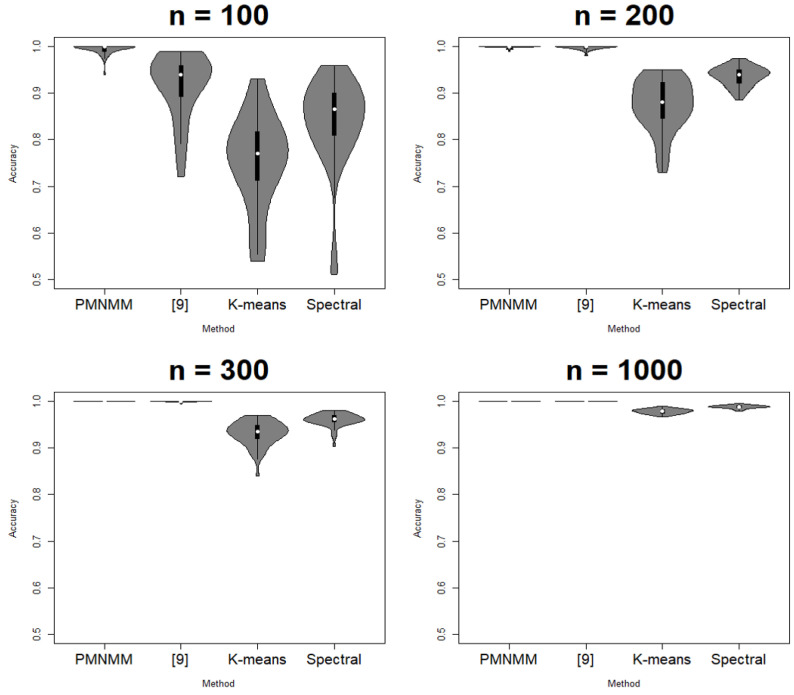
Violin plot of ACC in Scenario 1.

**Figure 4:**
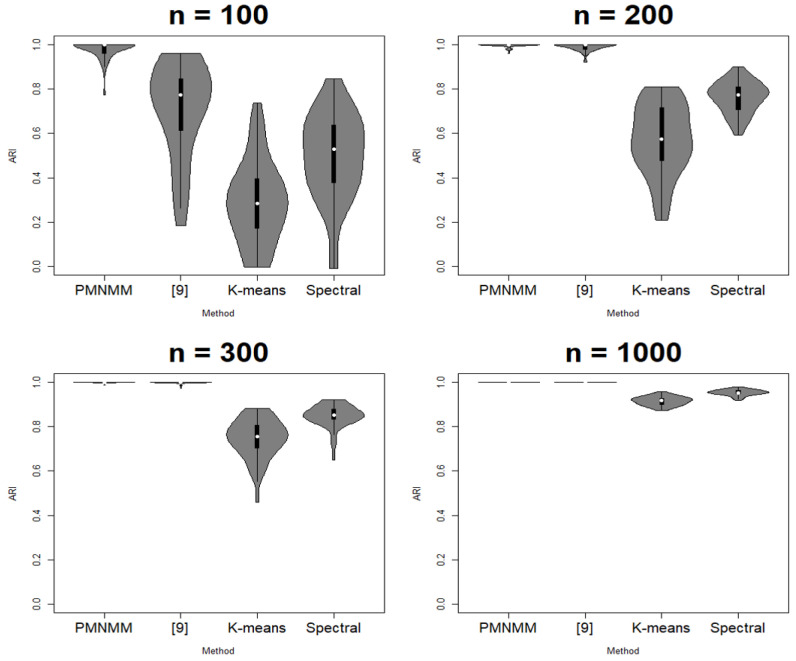
Violin plot of ARI in Scenario 1.

**Figure 5:**
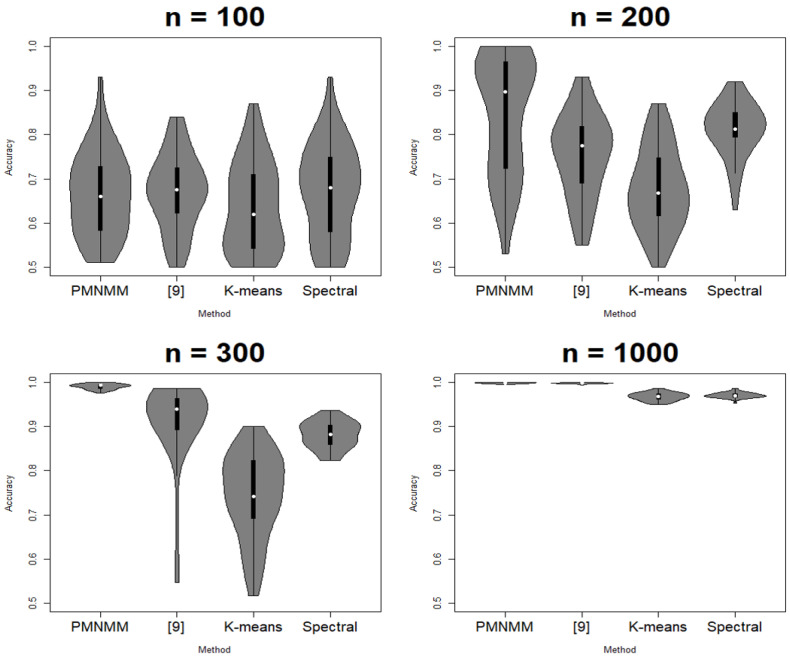
Violin plot of ACC in Scenario 2.

**Figure 6:**
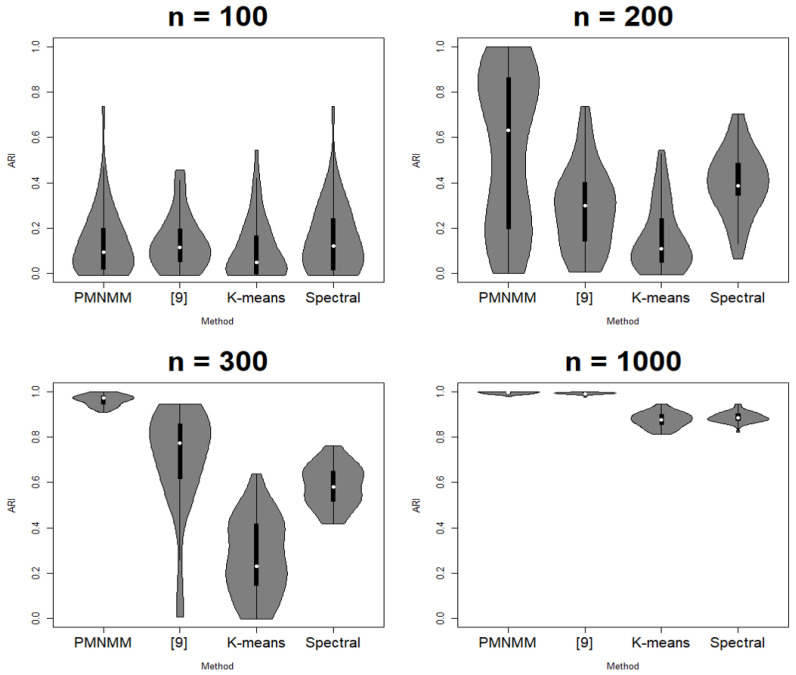
Violin plot of ARI in Scenario 2.

Since the clustering results depend on the generated data, each scenario is run 50 times. ACC, ARI, SL, and FL are calculated each time, and their average results are reported in Table 1, Table 2, Table 3 and Table 4, respectively. Figure 3, Figure 4, Figure 5 and Figure 6 show the violin plots of the ACC and ARI for each method with a box plot and underlying distribution by sample size in Scenario 1 and 2.

In Scenario 1, Figure 3 and Figure 4 and Table 1 show that the proposed model has the highest ACC (0.995) and ARI (0.98) for sample size 100; the method of [9] follows next. In addition, as n increases, the performance of PMNMM and the method of [9] become similar. To see if there is any significant difference among the means of the ACC and ARI, the Kruskal–Wallis test [22] is applied. The *p*-value of the test for significant difference among the means of ACC and ARI is 0, so we can confirm that the ACC and ARI means of the models differ under the significance level α=0.05. Then, to compare pairwise difference, the Dunn test [23] is applied. The null hypothesis of the Dunn test is that there is no difference between the two groups. As a result, PMNMM and the method of [9] is better than both K-means and the spectral method (*p*-value = 0), and the spectral method is better than K-means (*p*-value = 0) in all sample sizes. For a small sample case (n = 100), the proposed model shows better performance (*p*-value = 0) because there are many zero-valued elements of the precision matrix in the outside of the band, which are estimated to be relatively better than other methods. For the other sample size, *p*-values of the Dunn test between the ACC of PMNMM and the method of [9] is 0.9436, 1, and 1, respectively, and 0.8309, 1, 1 for ARI, respectively. Because the null hypothesis cannot be rejected, the performance of the two models is considered to be the same. As shown in Figure 3 and Figure 4, the ACC and ARI values of the K-means method, spectral method and the method of [9] are more spread than those of PMNMM. This implies that the proposed method produces more stable clustering results.

Table 2 shows the mean measured errors between estimated parameters and true parameters for 50 runs in Scenario 1. As the sample size increases, the FL value of the estimated mean matrix decreases, and similarly, the SL and FL values of the estimated precision matrix decrease. To see if there is any significant difference among values for each sample size, the Kruskal–Wallis test and the Dunn test are applied. With the Kruskal–Wallis test, the *p*-values for the means of FL (mean), SL (precision), and FL (precision) are 0, so the means of measured errors for three norms differ among different sample sizes under the significance level α=0.05. Next, with the Dunn test, we can confirm that the means of each error decrease as the sample size increases, as *p*-values are smaller than the significance level α=0.05 for two different increasing sample sizes. This implies that the proposed method has consistency.

In Scenario 2, similar to the previous process, we can check if there is any significant difference among the means of the ACC and ARI. However, under the AR(1) precision structure, the results are slightly different. For the sample size n = 100, the *p*-values of the Kruskal–Wallis test are 0.19 and 0.17 for ACC and ARI, respectively, so we cannot reject the null hypothesis that the means of the ACC and ARI for all models are the same. For the other sample sizes, the *p*-value of the Kruskal–Wallis test is 0, so the Dunn test is applied. For n = 200, the *p*-value of the Dunn test between PMNMM and spectral is 0.3911, so the null hypothesis cannot be rejected. On the contrary, the *p*-value of the Dunn test between PMNMM and the method of [9] is 0, so PMNMM is better than the method of [9]. The sparse precision matrix is not shown clearly for the data with n = 100 sample size in Scenario 2, and this hinders PMNMM from exploring sparsity in the precision matrix, showing similar performance as the conventional method. However, for n = 300, the *p*-values of the Dunn test between any pair of methods are 0. We can confirm that PMNMM is better than the K-means and spectral methods, as well as the method of [9]. For n = 1000, PMNMM and the method of [9] have the same performance (*p*-values = 1, 1 for ACC and ARI, respectively), and are better than the other two methods, but the performance of the other two methods is the same (*p*-values = 0.3433, 0.3294).

Table 4 shows the mean measured errors between estimated parameters and true parameters in scenario 2. Table 4 shows similar results as Table 2, and the Kruskal–Wallis test and the Dunn test are applied in the same way as before. As a result, for the Kruskal–Wallis test, the *p*-values for the test of significant difference among the means of each measured error are 0, and for the Dunn test, the *p*-values for all pairwise comparisons are 0, so all null hypotheses are rejected under the significance level α=0.05. This implies that the proposed method has consistency.

In the simulation studies, the two precision structures are considered: banding and AR(1). The experimental results of Scenario 1 showed that the performance of the proposed method is better than the other methods in all sample size cases; we can confirm the consistency of the proposed method through the measured errors with the increasing sample size. The penalized matrix normal mixture model in the banding precision matrix showed good performance, even with a very small sample size. However, in the AR(1) precision structure of Scenario 2, a larger number of samples was needed to achieve good performance, and the value of the measured error was higher when compared with Scenario 1. This implies that the proposed model works better for data with a more sparse precision structure.

### 3.2. Real Data Studies

- Datasets
  We evaluated the proposed model on the CIFAR 10 and Fashion-MNIST datasets:
  – CIFAR 10
    The CIFAR 10 dataset (https://www.cs.toronto.edu/~kriz/cifar.html, accessed on 21 September 2021) contains well-known image data. There are 60,000 images of 32 × 32 × 3 size with 10 types—airplane, automobile, bird, cat, deer, dog, frog, horse, ship, truck—and each type has 6000 images, respectively. The dataset is divided into a training set with 50,000 images and a test set with 10,000 images.
  – Fashion-MNIST
    The Fashion-MNIST dataset (https://github.com/zalandoresearch/fashion-mnist, accessed on 21 September 2021) is a grayscale image dataset, designed to replace the original MNIST dataset. There are 70,000 images of 28 × 28 size with 10 types—T-shirt/top, trouser, pullover, dress, coat, sandal, shirt, sneaker, bag, ankle boot— and each type has 7000 images, respectively. The dataset is divided into a training set with 60,000 images and a test set with 10,000 images.

#### 3.2.1. CIFAR 10 Dataset

**Figure 7:**
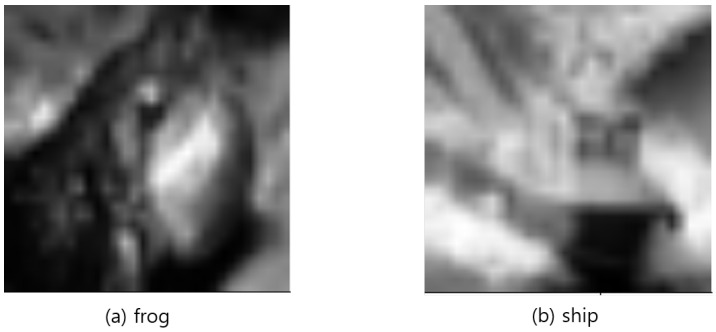
Two types of images in CIFAR 10 dataset: frog and ship.

In the experiment, two types of images are selected: frog and ship (Figure 7). For the performance comparison of the proposed method, the training images are transformed to grayscale, and 500 images are randomly selected from the training set for each type. Inaddition, the K-means and spectral methods, as well as the method of [9] are the conventional methods to be compared with PMNMM; the number of clusters G is set to 2; and the ranges of the tuning parameter $\lambda_{1},\lambda_{2},\lambda_{3}$ are set to $\lambda_{1}=1,0.5,0.1$, $\lambda_{2}=1,0.5,0.1,0.05,0.01$, $\lambda_{3}=1,0.5,0.1,0.05,0.01$. Using the training set, the optimal set of ($\lambda_{1},\lambda_{2},\lambda_{3}$) selected through the 4-fold CV method is (1, 0.01, 0.01), and the performance of the proposed model is evaluated on the testing set. A total of 500 images are randomly selected from the test set, and the models are fitted to obtain ACC and ARI, respectively. This process is repeated 50 times to calculate the average ACC and ARI of each model, and the results are shown in Table 5. Then, the Kruskal–Wallis test and the Dunn test are applied to see if there is any significant difference between the methods. As a result, the *p*-value of the Kruskal–Wallis test is 0, the ACC and ARI of the PMNMM are higher than those of K-means and spectral method with the Dunn test (*p*-value = 0), but PMNMM and the method of [9] have the same performance. The null hypothesis of the Dunn test between the K-means and spectral methods is not rejected (*p*-value = 0.075, 0.085 for ACC and ARI, respectively) under the significance level α=0.05.

To compare PMNMM with [9]’s method further, a more extreme low sample situation is set up by randomly selecting 10 images from the training set for each type. The results are shown in Table 6.

Table 5 and Table 6 show that the performance does not improve significantly even if the sample size increases because the two groups are difficult to be distinguished. There is also little performance difference between the two methods because the weight of the penalty imposed on the precision matrix is small.

#### 3.2.2. Fashion Dataset

**Figure 8:**
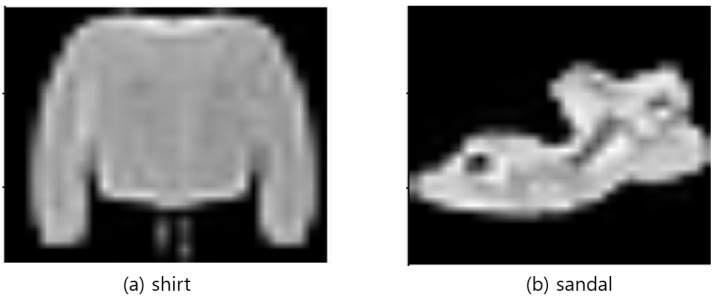
Two types of images in CIFAR 10 dataset: frog and ship.

We consider applying the methods to the fashion data, which can be distinguished more clearly. Similar to the CIFAR 10 data application, two types of images are selected—shirt and sandal—and 500 images are randomly selected from the training set for each type (Figure 8). The ranges of the tuning parameter $\lambda_{1},\lambda_{2},\lambda_{3}$ are set to $\lambda_{1}=1,0.5,0.1$, $\lambda_{2}=5,3,1,0.5,0.1$, $\lambda_{3}=5,3,1,0.5,0.1$. Using the training set, the optimal set of ($\lambda_{1},\lambda_{2},\lambda_{3}$) selected through the 4-fold CV method is (0.1, 0.1, 0.1). The results for ACC and ARI are shown in Table 7. It is confirmed that PMNMM and the method of [9] have almost the same performance for the sample size n = 1000. Then, the Kruskal–Wallis and Dunn tests are applied; the *p*-value of the Kruskal–Wallis test is 0; the ACC and ARI of the PMNMM are higher than those of K-means and spectral methods with the Dunn test (*p*-value = 0); and the null hypothesis of the Dunn test between the K-means and spectral methods is not rejected (*p*-value = 1) under the significance level α=0.05.

To compare PMNMM with the method of [9] further, a more extreme low sample situation is set up by randomly selecting 10 images from the training set for each type. The results are shown in Table 8.

Table 8 shows that the proposed method works better than the method of [9] that imposes a penalty only on the mean matrix for extremely small sample size data.

## 4. Conclusions

We have proposed a new penalized mixture model with the penalties imposed both on the mean matrix and precision matrix of the matrix normal mixture model. The conventional Gaussian mixture model for matrix data was challenging because it has to estimate one big covariance structure; therefore, the calculation speed becomes very slow as the dimension increases. On the contrary, the proposed method is a parsimonious model since it reduces a large number of parameters by regularizing the row and column variance matrices of the matrix normal distribution, especially imposing a lasso penalty on the precision matrix. Therefore, the proposed method can reflect the sparse conditional correlation structure and work even with high-dimensional data with a small sample size. The asymptotic distributions of the parameter estimators were derived, and their consistency was proved. We have shown the superior performance of the proposed model by comparing it with three conventional methods (method of [9], K-means method, and spectral method) through both synthetic and real data experiments. A performance comparison was done with ACC and ARI, and PMNMM was significantly better than the K-means and spectral methods by a nonparametric test. In the simulation studies, we evaluated the clustering performance for two different degree of sparsity of precision matrix. For the banding precision structure, the ACC performance of the proposed method reached 0.995 and 0.999, with the small sample size, n = 100 and 200, respectively, which are higher than the other methods. For the AR(1) precision structure, it is similar to that of [9] with n = 100 and higher than those of the other methods with n = 200, 300, and 1000. In conclusion, when the true precision matrix is sparse, that is, the variables have strong conditionally independent structure, the proposed model is well fitted to a small sample size data. For the real datasets, the ACC performance of the proposed method reaches 0.74 and 0.95 for clustering two types of CIFAR 10 and Fashion-MNIST images, respectively, which are higher than or equal to those of the other methods.

In the proposed model, calculation speed is an important factor, as mentioned above. Thus, the graphical lasso algorithm [12] was applied. A faster algorithm to estimate a large precision matrix would be beneficial for the proposed model to improve the calculation speed. In addition, to cluster a 3D image or higher dimensional data, new penalized mixture models for the tensor structure need to be developed. A penalized matrix non-Gaussian (such as skew-normal, skew-t) mixture model is worth developing for clustering more general types of matrix data.

## Figures and Tables

**Table 1:** Scenario 1: Performance comparison for PMNMM and conventional clustering methods (The values in parentheses are the standard deviations of the 50 runs’ results).

n	PMNMM		Method of [9]		K-Means		Spectral	
	ACC	ARI	ACC	ARI	ACC	ARI	ACC	ARI
100	0.995	0.98	0.915	0.706	0.754	0.288	0.838	0.489
	(0.01)	(0.041)	(0.07)	(0.213)	(0.097)	(0.184)	(0.097)	(0.198)
200	0.999	0.999	0.997	0.999	0.875	0.572	0.936	0.763
	(0.002)	(0.002)	(0.005)	(0.018)	(0.06)	(0.16)	(0.021)	(0.071)
300	1	1	1	1	0.932	0.747	0.961	0.849
	(0.0005)	(0.002)	(0.001)	(0.005)	(0.026)	(0.088)	(0.014)	(0.051)
1000	1	1	1	1	0.978	0.916	0.988	0.954
	(0.0002)	(0.0008)	(0.0002)	(0.0008)	(0.005)	(0.019)	(0.003)	(0.013)

**Table 2:** Scenario 1: Mean measured errors of the parameter estimates for the selected optimal parameter set (The values in parentheses are the standard deviations of the 50 runs’ results).

n	Optimal Parameter Set			FL	SL	FL
	$\lambda_{1}$	$\lambda_{2}$	$\lambda_{3}$	(Mean)	(Precision)	(Precision)
100	20	0.001	0.01	2.9	0.926	5.34
				(0.078)	(0.071)	(0.168)
200	0.1	0.01	0.1	2.19	0.597	3.9
				(0.048)	(0.045)	(0.088)
300	0.1	0.001	0.001	1.79	0.465	3.39
				(0.048)	(0.028)	(0.048)
1000	0.1	0.001	0.001	0.983	0.291	2.6
				(0.026)	(0.01)	(0.025)

**Table 3:** Scenario 2: Performance comparison for PMNMM and conventional clustering methods (The values in parentheses are the standard deviations of the 50 runs’ results).

n	PMNMM		Method of [9]		K-means		Spectral	
	ACC	ARI	ACC	ARI	ACC	ARI	ACC	ARI
100	0.665	0.135	0.671	0.137	0.636	0.105	0.674	0.155
	(0.094)	(0.146)	(0.086)	(0.122)	(0.1)	(0.132)	(0.104)	(0.155)
200	0.847	0.548	0.753	0.286	0.678	0.155	0.811	0.4
	(0.132)	(0.334)	(0.093)	(0.182)	(0.091)	(0.143)	(0.062)	(0.147)
300	0.991	0.964	0.909	0.705	0.743	0.269	0.881	0.583
	(0.006)	(0.024)	(0.097)	(0.227)	(0.095)	(0.168)	(0.03)	(0.092)
1000	0.999	0.994	0.998	0.993	0.968	0.876	0.971	0.888
	(0.001)	(0.005)	(0.001)	(0.004)	(0.009)	(0.032)	(0.006)	(0.022)

**Table 4:** Scenario 2: Mean measured errors of the parameter estimates for the selected optimal parameter set (The values in parentheses are the standard deviations of the 50 runs’ results).

n	Optimal Parameter Set			FL	SL	FL
	$\lambda_{1}$	$\lambda_{2}$	$\lambda_{3}$	(Mean)	(Precision)	(Precision)
100	0.1	0.001	0.1	4.51	1.23	6.77
				(0.253)	(0.155)	(0.372)
200	0.1	0.001	0.001	2.93	0.797	5.34
				(0.384)	(0.061)	(0.24)
300	0.1	0.001	0.001	2.02	0.621	4.71
				(0.062)	(0.047)	(0.054)
1000	0.1	0.001	0.001	1.11	0.422	4.11
				(0.026)	(0.013)	(0.027)

**Table 5:** Performance comparison of PMNMM with the conventional methods for CIFAR 10 dataset (The values in parentheses are the standard deviations of the 50 runs’ results).

Method	ACC	ARI
PMNMM	0.7418	0.2335
	(0.014)	(0.026)
[9]’s method	0.7418	0.2335
	(0.014)	(0.026)
K-means	0.6654	0.1088
	(0.015)	(0.019)
Spectral	0.6751	0.1222
	(0.017)	(0.023)

**Table 6:** Performance comparison of PMNMM and [9]’s method for CIFAR 10 dataset (The values in parentheses are the standard deviations of the 50 runs’ results).

Method	ACC	ARI
PMNMM	0.7206	0.1936
	(0.042)	(0.0753)
[9]’s method	0.7204	0.1934
	(0.043)	(0.0743)

**Table 7:** Performance comparison of PMNMM with the conventional methods for the fashion dataset (The values in parentheses are the standard deviations of the 50 runs’ results).

Method	ACC	ARI
PMNMM	0.9504	0.8113
	(0.007)	(0.024)
[9]’s method	0.9500	0.8095
	(0.014)	(0.026)
K-means	0.8828	0.5865
	(0.015)	(0.046)
Spectral	0.8831	0.5870
	(0.013)	(0.040)

**Table 8:** Performance comparison of PMNMM and the method of [9] for the fashion dataset (The values in parentheses are the standard deviations of the 50 runs’ results).

Method	ACC	ARI
PMNMM	0.9342	0.7541
	(0.025)	(0.086)
[9]’s method	0.6754	0.1207
	(0.026)	(0.037)

## Data Availability

The data presented in this study are available on https://www.cs.toronto.edu/~kriz/cifar.html, accessed on 21 September 2021 and https://github.com/zalandoresearch/fashion-mnist, accessed on 21 September 2021. The R code for the proposed method is available on request from the first author.

## Appendix A. A Comparative Overview Table

**Table A1:** A comparative overview table between the proposed method and the conventional methods.

	K-Means	Spectral	Gaussian Mixture	Mixture of Matrix Normal with Regularized Mean ([9])	Mixture of Matrix Normal with Regularized Mean/Covariance (Proposed Method)
Data type	Vector	Vector/Matrix	Vector	Matrix	Matrix
Clustering type	Centroid-based	Similarity-based	Model-based	Model-based	Model-based
Affine transformation invariance	X	X	O	O	O
Number of parameters (p×q data, number of clusters: G)	NA	NA	Gpq(pq+1)/2+ Gpq+G−1	Gp(p+1)/2+ Gq(q+1)/2+ Gpq+G−1−r	Gp(p+1)/2+ Gq(q+1)/2+ $Gpq+G-1-r^{*}$
Modeling ability for covariance structure of matrix data	X	X	X	O	O

*r*: the number of mean parameters values that are shrunken to zero (0≤r≤Gpq); $r^{*}$: the number of mean and precision parameters values that are shrunken to zero; $\left(r\leq r^{*}\leq Gp\left(p+1\right)/2+Gq\left(q+1\right)/2)+Gpq\right)$; NA: not applicable.

## Appendix B

**Proof**  **of Equation (5).**

$$\frac{\partial L_{p}\left(\Theta ;\Lambda \right)}{\partial M_{j}}=\frac{\partial }{\partial M_{j}}\left[\sum_{i=1}^{n}\tau_{ij}\left\{-\frac{1}{2}tr\left(V_{j}^{-1}{\left(X_{i}-M_{j}\right)}^{T}U_{j}^{-1}\left(x_{i}-M_{j}\right)\right)\right\}-\lambda_{1}{\left|M_{j}\right|}_{1}\right]=0,$$

using the formula $\frac{\partial }{\partial X}tr\left(AX^{T}BX\right)=BXA^{T}+B^{T}XA$ (KB Petersen, 2012 [24]), we obtain the following:

$$\begin{array}{cc} \; & \frac{\partial }{\partial M_{j}}\left[\sum_{i=1}^{n}\tau_{ij}\left\{-\frac{1}{2}tr\left(V_{j}^{-1}{\left(X_{i}-M_{j}\right)}^{T}U_{j}^{-1}\left(x_{i}-M_{j}\right)\right)\right\}-\lambda_{1}{\left|M_{j}\right|}_{1}\right]\\ \; & =\sum_{i=1}^{n}\tau_{ij}\left\{U_{j}^{-1}\left(X_{i}-M_{j}\right)V_{j}^{-1}\right\}-\lambda_{1}sign\left(M_{j}\right)=0. \end{array}$$

Some algebraic manipulations are applied as follows:

$$\begin{array}{cc} \; & \frac{\sum_{i=1}^{n}\tau_{ij}\left\{U_{j}^{-1}\left(X_{i}-M_{j}\right)V_{j}^{-1}\right\}}{\sum_{i=1}^{n}\tau_{ij}}-\frac{\lambda_{1}}{\sum_{i=1}^{n}\tau_{ij}}sign\left(M_{j}\right)=0.\\ \Rightarrow & \;U_{j}^{-1}\frac{\sum_{i=1}^{n}\tau_{ij}X_{i}}{\sum_{i=1}^{n}\tau_{ij}}V_{j}^{-1}-U_{j}^{-1}M_{j}V_{j}^{-1}-\frac{\lambda_{1}}{\sum_{i=1}^{n}\tau_{ij}}sign\left(M_{j}\right)=0.\\ \Rightarrow & \;\frac{\sum_{i=1}^{n}\tau_{ij}X_{i}}{\sum_{i=1}^{n}\tau_{ij}}-M_{j}-\frac{\lambda_{1}}{\sum_{i=1}^{n}\tau_{ij}}U_{j}sign\left(M_{j}\right)V_{j}=0. \end{array}$$

Because $\frac{\sum_{i=1}^{n}\tau_{ij}X_{i}}{\sum_{i=1}^{n}\tau_{ij}}$ is the estimate of $M_{j}$ for the non-penalty model, the following equation is derived:

(A1) $$\begin{array}{cc} {\hat{M}}_{j} & ={\tilde{M}}_{j}-\frac{\lambda_{1}}{\sum_{i=1}^{n}\tau_{ij}}U_{j}sign\left({\tilde{M}}_{j}\right)V_{j}\\ \; & =sign\left({\tilde{M}}_{j}\right)\left[|{\tilde{M}}_{j}|-\frac{\lambda_{1}}{\sum_{i=1}^{n}\tau_{ij}}U_{j}1_{p\times q}V_{j}\right]. \end{array}$$

□

## Appendix C

**Proof** **of Theorem 1.** Let $U_{j}^{*}$ and $V_{j}^{*}$ be the inverse matrix of $U_{j}$ and $V_{j}$, respectively. Then, the penalized *Q* function can be rewritten in the M-step as follows.

$$\begin{array}{cc} Q_{p}= & \sum_{j=1}^{G}\sum_{i=1}^{n}\left[-\frac{pq}{2}log\left(2\pi \right)+\frac{p}{2}log|V_{j}^{*}|+\frac{q}{2}log\left|U_{j}^{*}\right|-\frac{1}{2}tr\left\{V_{j}^{*}{\left(X_{i}-M_{j}\right)}^{T}U_{j}^{*}\left(X_{i}-M_{j}\right)\right\}\right]\\ \; & -\lambda_{1}\sum_{j=1}^{G}|M_{j}{|}_{1}-\lambda_{2}\sum_{j=1}^{G}|U_{j}^{*}{|}_{1}-\lambda_{3}\sum_{j=1}^{G}{\left|V_{j}^{*}\right|}_{1}. \end{array}$$

We took a similar approach as that which [4] used to derive the asymptotic distribution of the precision matrix estimator in the Gaussian graphical model.  □

### Appendix C.1. The Limiting Dist. of ${\tilde{M}}_{j}$

Let ${\tilde{M}}_{j}$ be the matrix that minimizes the following:

$$\begin{array}{c} \frac{1}{2}\sum_{i=1}^{n}\tau_{ij}tr\left\{V_{j}^{*}{\left(X_{i}-M_{j}\right)}^{T}U_{j}^{*}\left(X_{i}-M_{j}\right)\right\}+\lambda_{1}\left|{\tilde{M}}_{j}\right|,\;or\\ \sum_{i=1}^{n}\tau_{ij}tr\left\{V_{j}^{*}{\left(X_{i}-M_{j}\right)}^{T}U_{j}^{*}\left(X_{i}-M_{j}\right)\right\}/\sum_{i=1}^{n}\tau_{ij}+\lambda_{1}^{*}\left|{\tilde{M}}_{j}\right|, \end{array}$$

where $\lambda_{1}^{*}=2\lambda_{1}/\sum_{i=1}^{n}\tau_{ij}$.

Define $V_{1n}\left(H\right)$ as the following:

$$\begin{array}{cc} V_{1n}\left(H\right) & =\sum_{i=1}^{n}\tau_{ij}tr\left\{V_{j}^{*}{\left(X_{i}-M_{j}-H/\sqrt{n}\right)}^{T}U_{j}^{*}\left(X_{i}-M_{j}-H/\sqrt{n}\right)\right\}/\sum_{i=1}^{n}\tau_{ij}\\ \; & +\lambda_{1}^{*}\sum_{l=1}^{p}\sum_{m=1}^{q}\left|{\tilde{M}}_{j}\left(l,m\right)+H\left(l,m\right)/\sqrt{n}\right|\\ \; & -\sum_{i=1}^{n}\tau_{ij}tr\left\{V_{j}^{*}{\left(X_{i}-M_{j}\right)}^{T}U_{j}^{*}\left(X_{i}-M_{j}\right)\right\}/\sum_{i=1}^{n}\tau_{ij}+\lambda_{1}^{*}\sum_{l=1}^{p}\sum_{m=1}^{q}\left|{\tilde{M}}_{j}\left(l,m\right)\right|. \end{array}$$

Note the following:

$$\begin{array}{cc} \; & \sum_{i=1}^{n}\tau_{ij}tr\left\{V_{j}^{*}{\left(X_{i}-M_{j}-H/\sqrt{n}\right)}^{T}U_{j}^{*}\left(X_{i}-M_{j}-H/\sqrt{n}\right)\right\}/\sum_{i=1}^{n}\tau_{ij}\\ \; & -\sum_{i=1}^{n}\tau_{ij}tr\left\{V_{j}^{*}{\left(X_{i}-M_{j}\right)}^{T}U_{j}^{*}\left(X_{i}-M_{j}\right)\right\}/\sum_{i=1}^{n}\tau_{ij}\\ = & -tr\left\{V_{j}^{*}{\left(\sum_{i=1}^{n}\tau_{ij}\left(\frac{X_{i}-M_{j}}{\sum_{i=1}^{n}\tau_{ij}}\right)\right)}^{T}U_{j}^{*}\left(H/\sqrt{n}\right)\right\}-tr\left\{V_{j}^{*}{\left(H/\sqrt{n}\right)}^{T}U_{j}^{*}\left(\sum_{i=1}^{n}\tau_{ij}\left(\frac{X_{i}-M_{j}}{\sum_{i=1}^{n}\tau_{ij}}\right)\right)\right\}\\ \; & +tr(V_{j}^{*}H^{T}U_{j}^{*}H/\sqrt{n})\\ = & -tr\left\{U_{j}^{*}HV_{j}^{*}{\left({\hat{M}}_{j}-M_{j}\right)}^{T}\right\}/\sqrt{n}-tr\left\{V_{j}^{*}H^{T}U_{j}^{*}\left({\hat{M}}_{j}-M_{j}\right)\right\}/\sqrt{n}\\ \; & +tr\left(V_{j}^{*}H^{T}U_{j}^{*}H\right)/\sqrt{n}, \end{array}$$

where ${\hat{M}}_{j}=\sum_{i=1}^{n}\tau_{ij}X_{i}/\sum_{i=1}^{n}\tau_{ij}$, which is the m.l.e. of $M_{j}$ for the non-penalized matrix normal mixture model.

Together with the fact that

$$\begin{array}{cc} \; & \lambda_{1}^{*}\sum_{l=1}^{p}\sum_{m=1}^{q}\left(\right|{\tilde{M}}_{j}\left(l,m\right)+H\left(l,m\right)/\sqrt{n}\left|-\right|{\tilde{M}}_{j}\left(l,m\right)\left|\right)\\ \; & =\frac{\lambda_{1}^{*}}{\sqrt{n}}\sum_{l=1}^{p}\sum_{m=1}^{q}\left\{H\left(l,m\right)sign\left({\tilde{M}}_{j}\left(l,m\right)\right)I\left({\tilde{M}}_{j}\left(l,m\right)\neq 0\right)+\left|H\left(l,m\right)\right|I\left({\tilde{M}}_{j}\left(l,m\right)=0\right)\right\}, \end{array}$$

$nV_{1n}\left(H\right)$ can be rewritten as follows:

$$\begin{array}{cc} \; & -tr\left(U_{j}^{-1}HV_{j}^{-1}W_{1n}^{T}\right)-tr\left(V_{j}^{-1}H^{T}U_{j}^{-1}W_{1n}\right)+tr\left(V_{j}^{-1}H^{T}U_{j}^{-1}H\right)\\ \; & +\sqrt{n}\lambda_{1}^{*}\sum_{l=1}^{p}\sum_{m=1}^{q}\left\{H\left(l,m\right)sign\left({\tilde{M}}_{j}\left(l,m\right)\right)I\left({\tilde{M}}_{j}\left(l,m\right)\neq 0\right)+\left|H\left(l,m\right)\right|I\left({\tilde{M}}_{j}\left(l,m\right)=0\right)\right\}, \end{array}$$

where $W_{1n}=\sqrt{n}\left({\hat{M}}_{j}-M_{j}\right)$ and $vec\left(W_{1n}\right)\overset{d}{\to }N\left(0,\Lambda_{1}\right)$. Therefore, $nV_{1n}\left(H\right)\overset{d}{\to }V\left(H\right)$.

Since both $V_{1}\left(H\right)$ and $nV_{1n}\left(H\right)$ are convex and $V_{1}\left(H\right)$ has a unique minimum, we have the following:

(A2) $$argmin\;nV_{1n}\left(H\right)=\sqrt{n}\left({\tilde{M}}_{j}-M_{j}\right)\overset{d}{\to }argmin\;V_{1}\left(H\right)$$

### Appendix C.2. The Limiting Dist. of ${\tilde{U}}_{j}^{*}$

Let ${\tilde{U}}_{j}^{*}$ be the positive definite matrix that minimizes the following:

$$\begin{array}{c} -\frac{q\sum_{i=1}^{n}\tau_{ij}}{2}log|{\tilde{U}}_{j}^{*}|+\frac{q\sum_{i=1}^{n}\tau_{ij}}{2}tr\left({\tilde{U}}_{j}^{*}S_{j}^{\left(U\right)}\right)+\lambda_{2}{\left|{\tilde{U}}_{j}^{*}\right|}_{1}, \end{array}$$

where $S_{j}^{\left(U\right)}=\frac{\sum_{i=1}^{n}\tau_{ij}\left(X_{i}-M_{j}\right)V_{j}^{*}{\left(X_{i}-M_{j}\right)}^{T}}{q\sum_{i=1}^{n}\tau_{ij}}$, which is the m.l.e. of $U_{j}$ for the non-penalized matrix normal mixture model, or minimizes the following:

$$\begin{array}{c} -log|{\tilde{U}}_{j}^{*}|+tr\left({\tilde{U}}_{j}^{*}S_{j}^{\left(U\right)}\right)+\lambda_{2}^{*}{\left|{\tilde{U}}_{j}^{*}\right|}_{1}, \end{array}$$

where $\lambda_{2}^{*}=2\lambda_{2}/\left(q\sum_{i=1}^{n}\tau_{ij}\right)$.

Define $V_{2n}\left(L\right)$ as follows:

$$\begin{array}{cc} V_{2n}\left(L\right)= & -log|{\tilde{U}}_{j}^{*}+\frac{L}{\sqrt{n}}|+tr\left\{\left({\tilde{U}}_{j}^{*}+\frac{L}{\sqrt{n}}\right)S_{j}^{\left(U\right)}\right\}+\lambda_{2}^{*}\sum_{l\neq m}\left|{\tilde{U}}_{j}^{*}\left(l,m\right)+\frac{L(l,m)}{\sqrt{n}}\right|\\ \; & +log|{\tilde{U}}_{j}^{*}|-tr\left({\tilde{U}}_{j}^{*}S_{j}^{\left(U\right)}\right)+\lambda_{2}^{*}\sum_{l\neq m}\left|{\tilde{U}}_{j}^{*}\left(l,m\right)\right|. \end{array}$$

Note the following:

$$\begin{array}{c} log|{\tilde{U}}_{j}^{*}+\frac{L}{\sqrt{n}}\left|-log\right|{\tilde{U}}_{j}^{*}|=log|I+\frac{U_{j}^{1/2}LU_{j}^{1/2}}{\sqrt{n}}|=\sum_{i=1}^{p}log\left\{1+\sigma_{i}\left(U_{j}^{1/2}LU_{j}^{1/2}/\sqrt{n}\right)\right\}, \end{array}$$

where $\sigma_{j}\left(\cdot \right)$ denotes the *i*th-largest eigenvalue of a matrix. Since

$$\begin{array}{c} log\left\{1+\sigma_{i}\left(U_{j}^{1/2}LU_{j}^{1/2}/\sqrt{n}\right)\right\}=\frac{\sigma_{i}\left(U_{j}^{1/2}LU_{j}^{1/2}\right)}{\sqrt{n}}-\frac{\sigma_{i}^{2}\left(U_{j}^{1/2}LU_{j}^{1/2}\right)}{n}+o\left(\frac{1}{n}\right), \end{array}$$

we conclude the following:

$$\begin{array}{cc} log|{\tilde{U}}_{j}^{*}+\frac{L}{\sqrt{n}}\left|-log\right|{\tilde{U}}_{j}^{*}|= & \sum_{i=1}^{p}\frac{\sigma_{i}\left(U_{j}^{1/2}LU_{j}^{1/2}\right)}{\sqrt{n}}-\frac{tr(U_{j}^{1/2}LU_{j}LU_{j}^{1/2})}{n}+o\left(\frac{1}{n}\right)\\ \; & =\frac{tr(U_{j}^{1/2}LU_{j}^{1/2})}{\sqrt{n}}-\frac{tr(U_{j}^{1/2}LU_{j}LU_{j}^{1/2})}{n}+o\left(\frac{1}{n}\right)\\ \; & =\frac{tr(LU_{j})}{\sqrt{n}}-\frac{tr(LU_{j}LU_{j})}{n}+o\left(\frac{1}{n}\right). \end{array}$$

On the other hand,

$$\begin{array}{cc} tr\left\{\left({\tilde{U}}_{j}^{*}+\frac{L}{\sqrt{n}}\right)S_{j}^{\left(U\right)}\right\}-tr\left({\tilde{U}}_{j}^{*}S_{j}^{\left(U\right)}\right) & =tr(\frac{LS_{j}^{\left(U\right)}}{\sqrt{n}})\\ \; & =\frac{tr(LU_{j})}{\sqrt{n}}+\frac{tr\left\{L(S_{j}^{\left(U\right)}-U_{j})\right\}}{\sqrt{n}}. \end{array}$$

Together with the fact that

$$\begin{array}{cc} \; & \lambda_{2}^{*}\sum_{l\neq m}\left(|{\tilde{U}}_{j}^{*}\left(l,m\right)+\frac{L(l,m)}{\sqrt{n}}\right)\left|-\right|{\tilde{U}}_{j}^{*}\left(l,m\right)\left|\right)\\ \; & =\frac{\lambda_{2}^{*}}{\sqrt{n}}\sum_{l\neq m}\left\{L\left(l,m\right)sign\left({\tilde{U}}_{j}^{*}\left(l,m\right)\right)I\left({\tilde{U}}_{j}^{*}\left(l,m\right)\neq 0\right)+\left|L\left(l,m\right)\right|I\left({\tilde{U}}_{j}^{*}\left(l,m\right)=0\right)\right\}, \end{array}$$

$nV_{2n}\left(L\right)$ can be rewritten as follows:

$$\begin{array}{cc} \; & tr\left(LU_{j}LU_{j}\right)+tr\left(LW_{2n}\right)\\ \; & +\sqrt{n}\lambda_{2}^{*}\sum_{l\neq m}\left\{L\left(l,m\right)sign\left({\tilde{U}}_{j}^{*}\left(l,m\right)\right)I\left({\tilde{U}}_{j}^{*}\left(l,m\right)\neq 0\right)+\left|L\left(l,m\right)\right|I\left({\tilde{U}}_{j}^{*}\left(l,m\right)=0\right)\right\}+o\left(1\right), \end{array}$$

where $W_{2n}=\sqrt{n}\left(S_{j}^{\left(U\right)}-U_{j}\right)$ and $vec\left(W_{2n}\right)\overset{d}{\to }N\left(0,\Lambda_{2}\right)$ because $S_{j}^{\left(U\right)}$ is the m.l.e. of $U_{j}$ for non-penalized matrix normal mixture model. Since both $V_{2}\left(L\right)$ and $nV_{2n}\left(L\right)$ are convex and $V_{2}\left(L\right)$ has a unique minimum, the following holds:

(A3) $$argmin\;nV_{2n}\left(L\right)=\sqrt{n}\left({\tilde{U}}_{j}^{*}-U_{j}\right)\overset{d}{\to }argmin\;V_{2}\left(L\right)$$

### Appendix C.3. The Limiting Dist. of $V_{j}^{*}$

Let ${\tilde{V}}_{j}^{*}$ be the positive definite matrix that minimizes the following:

$$\begin{array}{c} -\frac{p\sum_{i=1}^{n}\tau_{ij}}{2}log|{\tilde{V}}_{j}^{*}|+\frac{p\sum_{i=1}^{n}\tau_{ij}}{2}tr\left({\tilde{V}}_{j}^{*}S_{j}^{\left(V\right)}\right)+\lambda_{3}{\left|{\tilde{V}}_{j}^{*}\right|}_{1}, \end{array}$$

where $S_{j}^{\left(V\right)}=\frac{\sum_{i=1}^{n}\tau_{ij}{\left(X_{i}-M_{j}\right)}^{T}U_{j}^{*}\left(X_{i}-M_{j}\right)}{p\sum_{i=1}^{n}\tau_{ij}}$, or minimizes the following:

$$\begin{array}{c} -log|{\tilde{V}}_{j}^{*}|+tr\left({\tilde{V}}_{j}^{*}S_{j}^{\left(V\right)}\right)+\lambda_{3}^{*}{\left|{\tilde{V}}_{j}^{*}\right|}_{1}, \end{array}$$

where $\lambda_{3}^{*}=2\lambda_{3}/\left(p\sum_{i=1}^{n}\tau_{ij}\right)$.

Define $V_{3n}\left(R\right)$ as follows:

$$\begin{array}{cc} V_{3n}\left(R\right)= & -log|{\tilde{V}}_{j}^{*}+\frac{R}{\sqrt{n}}|+tr\left\{\left({\tilde{V}}_{j}+\frac{R}{\sqrt{n}}\right)S_{j}^{\left(V\right)}\right\}+\lambda_{3}^{*}\sum_{l\neq m}\left|{\tilde{V}}_{j}^{*}\left(l,m\right)+\frac{R(l,m)}{\sqrt{n}}\right|.\\ \; & +log|{\tilde{V}}_{j}^{*}|-tr\left({\tilde{V}}_{j}^{*}S_{j}^{\left(V\right)}\right)+\lambda_{3}^{*}\sum_{l\neq m}\left|{\tilde{V}}_{j}^{*}\left(l,m\right)\right| \end{array}$$

Note the following:

$$\begin{array}{c} log|{\tilde{V}}_{j}^{*}+\frac{R}{\sqrt{n}}\left|-log\right|{\tilde{V}}_{j}^{*}|=log|I+\frac{V_{j}^{1/2}RV_{j}^{1/2}}{\sqrt{n}}|=\sum_{i=1}^{q}log\left\{1+\sigma_{i}\left(V_{j}^{1/2}RV_{j}^{1/2}/\sqrt{n}\right)\right\}. \end{array}$$

Since

$$\begin{array}{c} log\left\{1+\sigma_{i}\left(V_{j}^{1/2}RV_{j}^{1/2}/\sqrt{n}\right)\right\}=\frac{\sigma_{i}\left(V_{j}^{1/2}RV_{j}^{1/2}\right)}{\sqrt{n}}-\frac{\sigma_{i}^{2}\left(V_{j}^{1/2}RV_{j}^{1/2}\right)}{n}+o\left(\frac{1}{n}\right), \end{array}$$

we conclude the following:

$$\begin{array}{cc} log|{\tilde{V}}_{j}^{*}+\frac{R}{\sqrt{n}}\left|-log\right|{\tilde{V}}_{j}^{*}|= & \sum_{i=1}^{q}\frac{\sigma_{i}\left(V_{j}^{1/2}RV_{j}^{1/2}\right)}{\sqrt{n}}-\frac{tr(V_{j}^{1/2}RV_{j}RV_{j}^{1/2})}{n}+o\left(\frac{1}{n}\right)\\ \; & =\frac{tr(V_{j}^{1/2}RV_{j}^{1/2})}{\sqrt{n}}-\frac{tr(V_{j}^{1/2}RV_{j}RV_{j}^{1/2})}{n}+o\left(\frac{1}{n}\right)\\ \; & =\frac{tr(RV_{j})}{\sqrt{n}}-\frac{tr(RV_{j}RV_{j})}{n}+o\left(\frac{1}{n}\right). \end{array}$$

On the other hand,

$$\begin{array}{cc} tr\left\{\left({\tilde{V}}_{j}^{*}+\frac{R}{\sqrt{n}}\right)S_{j}^{\left(V\right)}\right\}-tr\left({\tilde{V}}_{j}^{*}S_{j}^{\left(V\right)}\right) & =tr(\frac{RS_{j}^{\left(V\right)}}{\sqrt{n}})\\ \; & =\frac{tr(RV_{j})}{\sqrt{n}}+\frac{tr\left\{R(S_{j}^{\left(V\right)}-V_{j})\right\}}{\sqrt{n}}. \end{array}$$

Together with the fact that

$$\begin{array}{cc} \; & \lambda_{3}^{*}\sum_{l\neq m}\left(|{\tilde{V}}_{j}^{*}\left(l,m\right)+\frac{R(l,m)}{\sqrt{n}}\right)\left|-\right|{\tilde{V}}_{j}^{*}\left(l,m\right)\left|\right)\\ \; & =\frac{\lambda_{3}^{*}}{\sqrt{n}}\sum_{l\neq m}\left\{R\left(l,m\right)sign\left({\tilde{V}}_{j}^{*}\left(l,m\right)\right)I\left({\tilde{V}}_{j}^{*}\left(l,m\right)\neq 0\right)+\left|R\left(l,m\right)\right|I\left({\tilde{V}}_{j}^{*}\left(l,m\right)=0\right)\right\}, \end{array}$$

$nV_{3n}\left(R\right)$ can be rewritten as follows:

$$\begin{array}{cc} \; & tr\left(RV_{j}RV_{j}\right)+tr\left(RW_{3n}\right)\\ \; & +\sqrt{n}\lambda_{3}^{*}\sum_{l\neq m}\left\{R\left(l,m\right)sign\left({\tilde{V}}_{j}^{*}\left(l,m\right)\right)I\left({\tilde{V}}_{j}^{*}\left(l,m\right)\neq 0\right)+\left|R\left(l,m\right)\right|I\left({\tilde{V}}_{j}^{*}\left(l,m\right)=0\right)\right\}+o\left(1\right), \end{array}$$

where $W_{3n}=\sqrt{n}\left(S_{j}^{\left(V\right)}-V_{j}\right)$ and $vec\left(W_{3n}\right)\overset{d}{\to }N\left(0,\Lambda_{3}\right)$ because $S_{j}^{\left(V\right)}$ is the m.l.e. of $V_{j}$ for non-penalized matrix normal mixture model. Since both $V_{3}\left(R\right)$ and $nV_{3n}\left(R\right)$ are convex and $V_{3}\left(R\right)$ has a unique minimum, we have the following:

(A4) $$argmin\;nV_{3n}\left(R\right)=\sqrt{n}\left({\tilde{V}}_{j}^{*}-V_{j}\right)\overset{d}{\to }argmin\;V_{3}\left(R\right)$$
